# Successful Percutaneous Ultrasonic Lithotripsy of Gallstones

**DOI:** 10.31486/toj.23.0071

**Published:** 2024

**Authors:** Adam Ostergar, Muhammad Hassan Alkazemi, Hayden Hill, Michael M. Awad, Alexander Ushinsky, Michael Darcy, Alana C. Desai, Robert Sherburne Figenshau

**Affiliations:** ^1^ Division of Urologic Surgery, Washington University School of Medicine in St. Louis, St. Louis, MO; ^2^ Department of Surgery, Washington University School of Medicine in St. Louis, St. Louis, MO; ^3^ Mallinckrodt Institute of Radiology, Washington University School of Medicine in St. Louis, St. Louis, MO

**Keywords:** *Cholelithiasis*, *endoscopy–digestive system*, *gallbladder diseases*, *gallstones*, *lithotripsy*, *ultrasonics*

## Abstract

**Background:** Acute calculous cholecystitis is the obstruction of the cystic duct by a gallstone that leads to inflammation of the gallbladder necessitating cholecystectomy.

**Case Series:** We present the cases of 2 patients with acute calculous cholecystitis who were deemed ineligible candidates for cholecystectomy because of their complicating medical histories. Both patients initially underwent cholecystostomy and drain placement with interventional radiology for management of acute calculous cholecystitis. Their large gallstones remained refractory to attempts at removal by electrohydraulic lithotripsy via the cholecystostomy access. The patients’ gallstones were successfully removed via percutaneous ultrasonic lithotripsy during a collaborative procedure with interventional radiology and urology.

**Conclusion:** An interdisciplinary approach using percutaneous cholecystolithotomy with rigid ultrasonic lithotripsy is an effective method for removing challenging gallstones in patients for whom traditional approaches fail.

## INTRODUCTION

Acute calculous cholecystitis—the obstruction of the cystic duct by a gallstone that leads to inflammation of the gallbladder—may present with right upper quadrant pain, nausea, vomiting, fever and, if left untreated, can result in perforation, abscess formation, or fistulae.^1^ The optimal treatment for acute calculous cholecystitis is surgical removal of the gallbladder by laparoscopic or conventional surgery.^2^ However, several studies show significant increases in postoperative mortality rates for otherwise healthy patients with medical comorbidities or advanced age.^3,4^ For these patients, an alternative treatment for acute or chronic cholecystitis is percutaneous cholecystolithotomy.^5^ Percutaneous cholecystolithotomy is similar to percutaneous nephrolithotomy, a procedure used for complex urinary stone disease, typically renal calculi >2 cm.

We present the cases of 2 medically comorbid patients who underwent percutaneous cholecystolithotomy for acute calculous cholecystitis. The patients’ stones proved refractory to removal via repeated electrohydraulic lithotripsy performed by interventional radiology. Electrohydraulic lithotripsy uses a charge generator that transmits across the electrodes of a bipolar probe, resulting in spark creation, expansion of fluid, and an oscillating shock wave of pressure that can fragment stones. Irrigation is used to transmit the shock wave, improve visualization, and flush away fragments as they are created.^6^

A cooperative approach between interventional radiology and urology with the use of the Swiss LithoClast Trilogy Lithotripter (E.M.S. Electro Medical Systems S.A.) provided effective treatment for both patients. In addition to its suction capability, this lithotripter uses a dual-energy system that generates both ultrasonic vibrations and electromagnetic energy for stone treatment. Suction was performed through Amplatz type renal dilators (Boston Scientific Corporation).

## CASE SERIES

### Case 1

A 53-year-old male presented for percutaneous cholecystolithotomy for a large recalcitrant gallstone. He had been hospitalized 1 year prior for acute cholecystitis. At that time, he underwent percutaneous drainage of the gallbladder as he was not considered an operative candidate because of severe respiratory disease and morbid obesity. The patient subsequently underwent serial catheter exchanges and 5 attempts at percutaneous stone extraction by interventional radiology; however, a large stone burden remained. Given the difficulty in fracturing his stone with electrohydraulic lithotripsy, urology was consulted for attempted ultrasonic lithotripsy.

Monitored anesthesia care with intravenous sedation was administered, and the patient was prepped around the existing cholecystostomy tube. Interventional radiology performed serial dilation of the preexisting access tract (16 French [F]) to accommodate a 30F sheath. The urology team used a rigid scope with an ultrasonic probe to enter the gallbladder. Multiple stones were visualized, fragmented, and evacuated with the ultrasonic lithotripter. When stone clearance was achieved, a catheter was advanced into the gallbladder through the sheath, contrast was injected to confirm intraluminal positioning, and a guidewire was coiled within the gallbladder lumen. The sheath was removed, and a new 16F drain was replaced in the gallbladder; position and bile duct patency were confirmed by contrast injection and fluoroscopy.

At 1-week interventional radiology follow-up, the patient had a patent cystic duct and no residual cholelithiasis, so the catheter was removed. The patient remained recurrence-free at his 4-month follow-up.

### Case 2

A 63-year-old male was admitted for an episode of acute cholecystitis and underwent percutaneous cholecystolithotomy with placement of a 14F catheter as he was deemed a poor surgical candidate because of recently diagnosed nonalcoholic steatohepatitis cirrhosis. Follow-up cholangiogram 2 months later showed persistent cholelithiasis. Interventional radiology attempted percutaneous stone removal with electrohydraulic lithotripsy. After 2 hours and the use of several electrohydraulic lithotripsy probes, only a small portion of the first stone had been fragmented, leaving a considerable amount of stone burden. The cholecystostomy tube was upsized to a 16F catheter, and plans were made to collaborate with urology for percutaneous ultrasonic lithotripsy.

**Figure 1.**
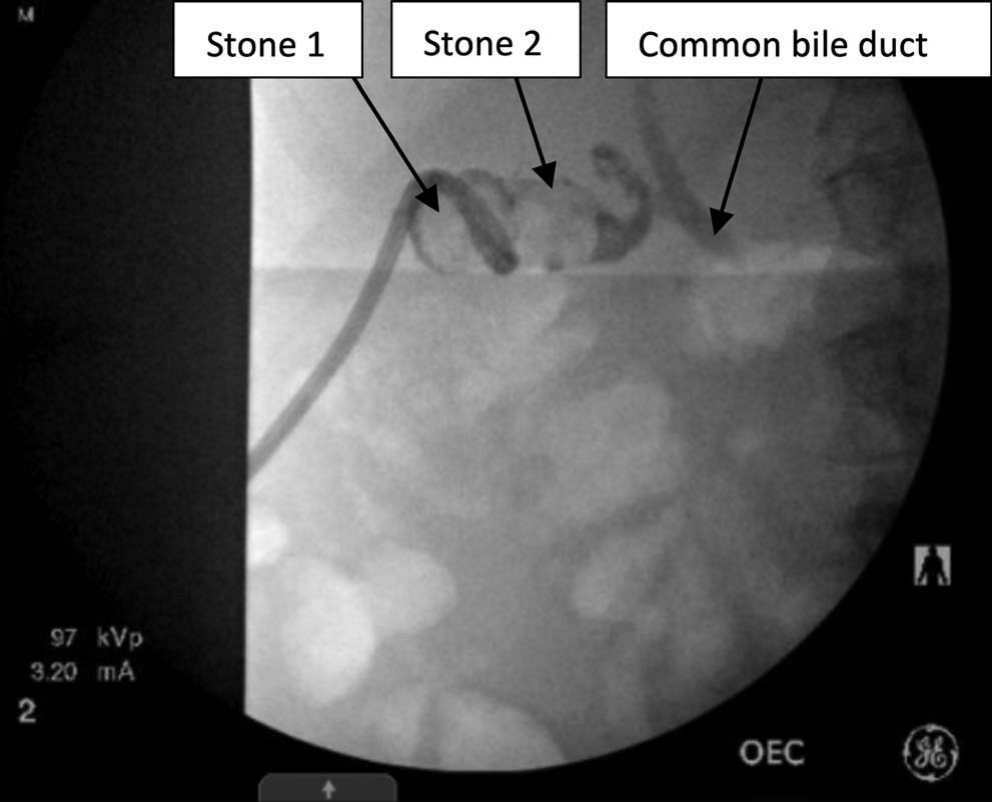
Case 2: Two large stones were visualized with fluoroscopy within the patient's gallbladder.

**Figure 2.**
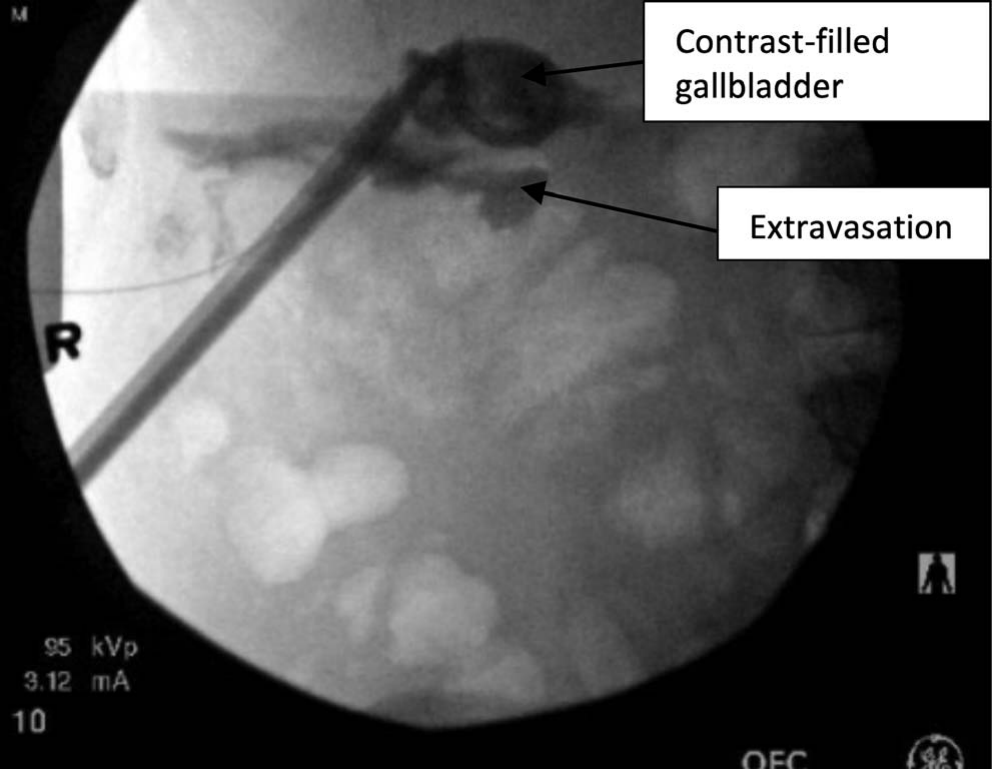
Case 2: Fluoroscopy shows the stones removed, the catheter in place, and some extravasation of contrast, indicating partial tract disruption.

Under general anesthesia, the access tract was dilated and a 24F sheath was placed. The urologist identified the first stone (Figure 1) and fragmented it with ultrasonic lithotripsy. Large fragments were removed with a basket, and small fragments were suctioned with the lithotripter. The second stone was identified and removed in similar fashion. The cystic duct was visualized and noted to be patent with efflux of bile. After successful ultrasound lithotripsy, a 16F catheter was placed within the gallbladder, and its position was confirmed under fluoroscopy. Some contrast extravasation from the gallbladder was seen, indicating partial disruption of the tract (Figure 2). The patient did not exhibit any pain or signs of peritonitis during the postoperative period.

**Figure 3.**
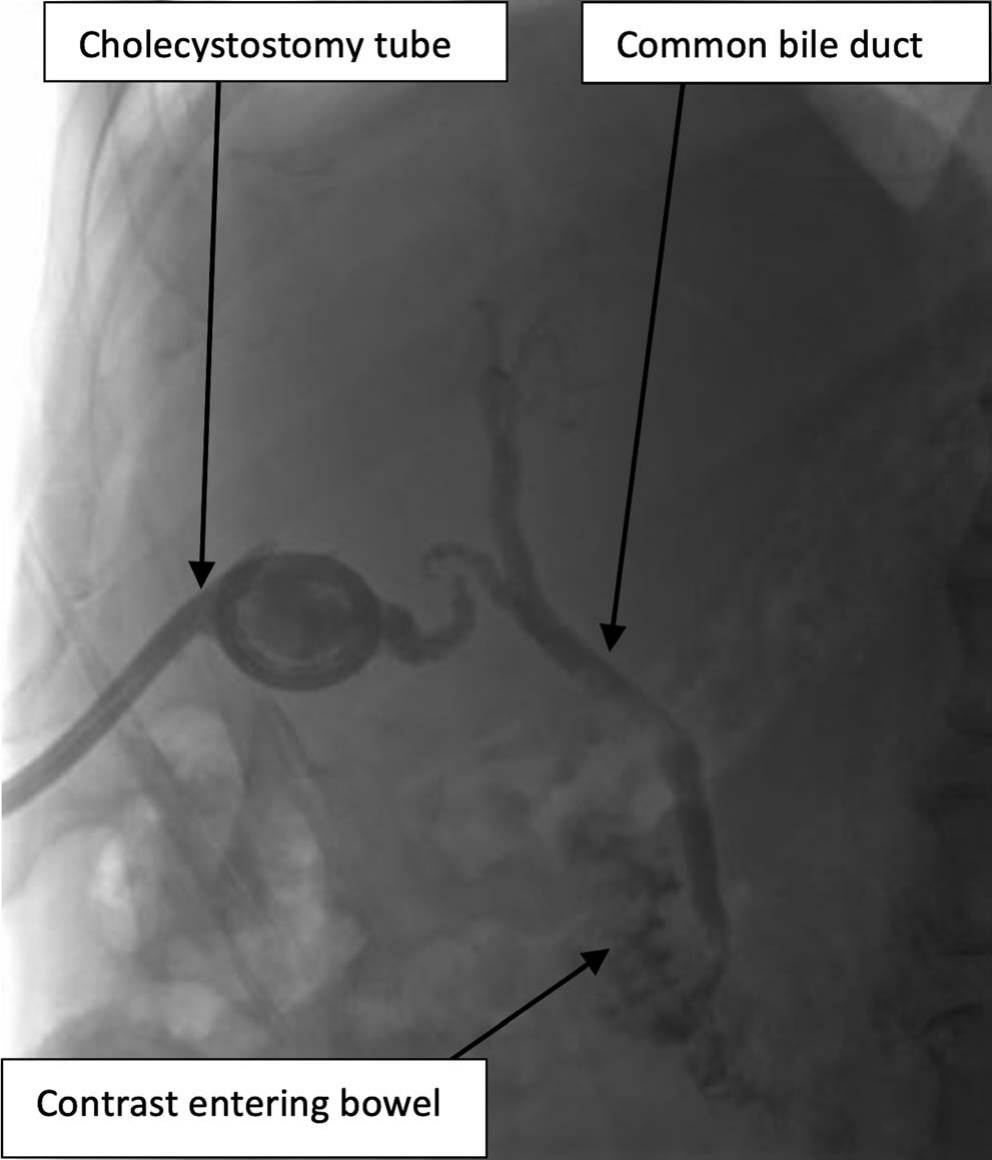
Case 2: Interval cholangiogram prior to cholecystostomy tube removal shows patency of the common bile duct, as contrast reaches the small bowel.

Interval biliary drainage catheter check performed 1 week later showed no residual stones or filling defects, and the cholecystostomy tube was removed (Figure 3). At 2-month follow-up, the patient remained asymptomatic.

## DISCUSSION

While laparoscopic cholecystectomy is the established procedure for patients with gallstones and/or acute cholecystitis,^2^ percutaneous cholecystolithotomy can be performed with less risk than laparoscopic cholecystectomy in patients with serious comorbidities.^3,7^ Persistent drainage through a cholecystostomy catheter causes discomfort, increased hospital visits for tube complications, and overall reduced quality of life. Successful percutaneous cholecystolithotomy can eliminate stones and symptoms and reduce recurrence and the need for drainage catheters. Percutaneous cholecystolithotomy can be used in patients with contraindications to general anesthesia (Case 1) or contraindications to surgery (Case 2). Although percutaneous cholecystolithotomy has been reported since the 1970s, its incidence has been slowly growing.^8^ Percutaneous cholecystolithotomy can be performed with low-profile flexible endoscopic devices and electrohydraulic, ultrasonic, or laser lithotripsy. As demonstrated in our patients, a collaborative approach between interventional radiology and urology should be considered for difficult stones. Continued monitoring of patients for recurrence of stones or future cholecystitis will help to determine the benefits of percutaneous cholecystolithotomy.

## CONCLUSION

These cases demonstrate that percutaneous cholecystolithotomy with rigid ultrasonic lithotripsy is an effective method for removing challenging gallstones in patients for whom traditional approaches fail.

## Notes

*This article meets the Accreditation Council for Graduate Medical Education and the American Board of Medical Specialties Maintenance of Certification competencies for Patient Care and Medical Knowledge.*
